# Dynamic Surface Tension Enhances the Stability of Nanobubbles in Xylem Sap

**DOI:** 10.3389/fpls.2021.732701

**Published:** 2021-12-16

**Authors:** Stephen Ingram, Yann Salmon, Anna Lintunen, Teemu Hölttä, Timo Vesala, Hanna Vehkamäki

**Affiliations:** ^1^Institute for Atmospheric and Earth System Research/Physics, University of Helsinki, Helsinki, Finland; ^2^Institute for Atmospheric and Earth System Research/Forest Sciences, University of Helsinki, Helsinki, Finland; ^3^Laboratory of Ecosystem-Atmospheric Interactions of Forest – Mire Complexes, Yugra State University, Khanty-Mansiysk, Russia

**Keywords:** tree hydraulics, molecular simulation (molecular modeling), nanobubbles, lipid monolayers, Classical Nucleation Theory (CNT)

## Abstract

Air seeded nanobubbles have recently been observed within tree sap under negative pressure. They are stabilized by an as yet unidentified process, although some embolize their vessels in extreme circumstances. Current literature suggests that a varying surface tension helps bubbles survive, but few direct measurements of this quantity have been made. Here, we present calculations of dynamic surface tension for two biologically relevant lipids using molecular dynamics simulations. We find that glycolipid monolayers resist expansion proportionally to the rate of expansion. Their surface tension increases with the tension applied, in a similar way to the viscosity of a non-Newtonian fluid. In contrast, a prototypical phospholipid was equally resistant to all applied tensions, suggesting that the fate of a given nanobubble is dependent on its surface composition. By incorporating our results into a Classical Nucleation Theory (CNT) framework, we predict nanobubble stability with respect to embolism. We find that the metastable radius of glycolipid coated nanobubbles is approximately 35 nm, and that embolism is in this case unlikely when the external pressure is *less negative* than –1.5 MPa.

## Introduction

Trees are capable of transporting water at high flow rates against gravity, without the aid of a mechanical pump like the heart in animals. Water vapor transpires from the leaves, generating tension (Pickard, 1981; Zimmermann et al., 1994), which pulls water further upwards in the xylem. This mechanism has come to be known as the cohesion-tension theory (Pockman et al., 1995). Severe tension or, more accurately, negative pressure, may disrupt the hydrogen bonds linking water molecules to one another, in much the same way that exposure to vacuum boils liquid water (Vera et al., 2016).

Nanobubbles further complicate the picture: it was recently discovered that gas-filled bubbles, tens to hundreds of nanometers in radius, can be “air seeded” into the liquid xylem sap at pit membranes (Schenk et al., 2015; Kaack et al., 2019). Intuitively, one would expect nanobubbles to be unstable with respect to “boiling” at highly negative pressures. Consider that a bubble’s enthalpy, *H*, can be decomposed into its internal energy, *U*, and the mechanical work required to expand the bubble volume, *v*(*r*), at radius *r*, against the external pressure, *p*:


(1) H⁢(r)=U⁢(r)+p⁢v⁢(r)

We can see that when the external pressure is negative, the mechanical work of bubble formation is also negative. Therefore, the enthalpy of formation is decreased (Menzl and Dellago, 2016) relative to a positive pressure, and the growth of existing bubbles stabilizes the system energetically (see also Supplementary Material).

Classical Nucleation Theory (CNT) tells us that creating or maintaining a gas-liquid interface requires enthalpy, whereas increasing the bubble volume maximizes entropy. The critical radius of a bubble is the size that balances these two contributions: Surface contributions dominate at small radii, below the critical size, and volume contributions above. Any bubble exceeding the critical radius should undergo runaway growth, forcing dissolved gas from the liquid phase into the gas phase and embolizing the xylem conduit surrounding it. Yet this is not the case: Tree sap has been reported to be more stable under tension than water (Schenk et al., 2017; Yang et al., 2020).

Recent experiments (Ohgaki et al., 2010) on nitrogen nanobubbles, prepared under standard conditions, have revealed that they can sustain positive internal pressures of at least 6 MPa (60 atmospheres) and remain stable. Only very recently have theoretical explanations of this phenomena (Manning, 2020) been attempted, and it remains unclear how nanobubbles are prevented from expanding into embolisms. On a more fundamental level, perhaps it is pertinent to consider whether the presence of a small number of nanobubbles within xylem sap may actually be beneficial for trees.

To our knowledge, the only computational study of the effects of negative pressure on a biological system thus far has been that of Kanduč et al. (2020) Bilayers of amphiphilic lipids were subjected to very high tensions (−20 MPa) and were found to cavitate (nucleate pockets of vacuum) at rates predictable by CNT. In addition, Molecular Dynamics (MD) studies of pure water (Abascal et al., 2013; Gonzalez et al., 2015) have found that pressures more negative than –100 MPa are required to promote cavitation at an observable rate. Such conditions are too extreme to be relevant to tree sap: Cavities will not form spontaneously in xylem.

It has been suggested that nanobubbles will rapidly become coated in lipids as they “bud off” from the pit membrane structure (Schenk et al., 2017). Their presence is significant: As Schenk and co-workers have pointed out (Schenk et al., 2017; Yang et al., 2020), the dynamic surface tension of a surfactant coated gas-liquid interface, under tension, is unknown. Nonetheless, the structural changes undergone by monolayers as they are stretched at positive pressures are well established (Duncan and Larson, 2008). We predict that phase transitions within the nanobubble coating are common at the pressures found in trees. Furthermore, we hypothesize that their surface tension will change as different monolayer morphologies are adopted (Wüstneck et al., 2005).

In this study, we have used MD simulations to investigate the dynamical behavior of air-lipid-water interfaces as they are stretched at biologically relevant negative pressures. The interfaces are flat and smaller than the internal surface area of a prototypical nanobubble, creating a “toy” system free from the effects of curvature, or bubble dissolution. We have produced pressure—area isotherms, which express the dynamic nature of surface tension as a function of the instantaneous area per lipid. Finally, we directly relate this quantity to the Gibbs free energy landscape of nanobubbles, predicting the pressure range within which embolism is likely.

## Materials and Methods

### Simulation Setup

All molecular dynamics simulations described herein were conducted using the 2020.2 iteration of GROMACS (Hess et al., 2008) running on the Puhti supercomputer at the CSC. GPU acceleration was provided using the CUDA platform (Kutzner et al., 2015), and each simulation utilized two NVIDIA Tesla v100 cards.

**FIGURE 1:**
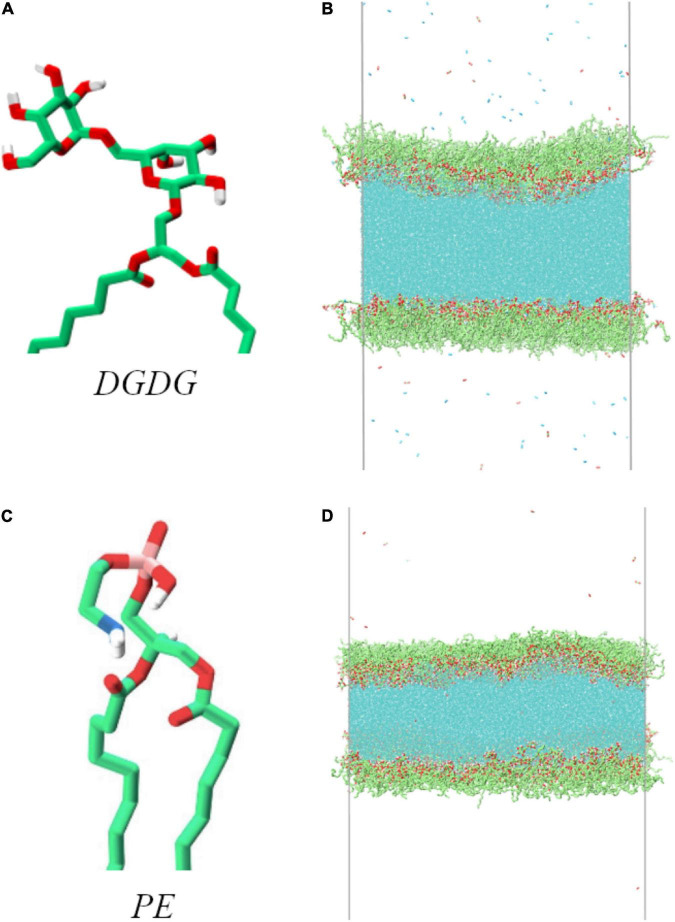
Head group structure of the two lipids used in this work, alongside atomistic representations of the equilibrated simulation boxes: digalactosyldiacylglycerol (DGDG, **A,B**) and phosphatidyl ethanolamine (PE, **C,D**). Carbon atoms and methylene groups are colored green, oxygen red, hydrogen white, nitrogen blue and phosphorus pink. Water molecules are colored cyan in **(B,D)**.

The simulations conducted consist of a horizontal slab of water coated with two monolayers of lipids decorating its top and bottom sides (in the *z* dimension). The lipids investigated were digalactosyldiacylglycerol (DGDG) and phosphatidyl ethanolamine (PE). The structure of their head groups can be seen in Figure 1. The two tail groups, extending below the bottom of the figure, are palmitic acid esters. The two gas phase volumes above and below the monolayers were filled with O_2_ and CO_2_ in a 50:50 ratio (for the DGDG) and N_2_, O_2_, and CO_2_ in an 80:10:10 ratio (for PE), at pressures of 0.02 and 0.1 MPa, respectively (approximately 1 molecule per 50 nm^3^). The difference in gas phase molecules included is not thought to affect the results at this pressure, as none of them were observed to reside to the water-lipid interface.

The major advantage of a double interface configuration is that any quantity we are interested in extracting (in this case surface tension and rupture area) can be determined for each monolayer separately, doubling the data extracted per simulation and reducing uncertainties in the desired quantities.

Our simulations were conducted in the *NpT* thermodynamic ensemble, which is to say that the number of atoms, their mean temperature, and the applied pressure are conserved over time. For both lipid systems, we have used a temperature of 298 K. We aim to understand the effect that different pulling rates have on the predicted nanobubble stability, beginning with the integrity of the interface itself.

The molecular configurations of the lipids were downloaded from the Limonada website.^1^ To increase simulation efficiency, both lipids were included as united atom molecules: all hydrogen atoms present in the tail groups were omitted and their masses and charges folded into (“united” with) the appropriate carbon atoms.

Both lipids studied here were simulated using a variant of the GROMOS 53a6 force field, described by van Eerden et al. (2015). Water was represented by the TIP4P-2005 model, which effectively replicates the experimental properties of bulk water (Vega and de Miguel, 2007; Abascal and Vega, 2010). In the gas phase, CO_2_ was represented by the EPM2 model (Cygan et al., 2012), and O_2_ and N_2_ by assigning the atoms the appropriate GROMOS atom type. The topology files used for both gas phase molecules were downloaded from the Automated Topology Builder^2^ (Koziara et al., 2014).

With regards to pressure coupling, the Berenden barostat was used semi-isotropically, i.e., acting only in the lateral *xy* plane. The size of the box in the *z* dimension (thickness of the gas phase) is kept constant (60 nm for DGDG simulations, 45 nm for PE). The lateral compressibility of the all membranes investigated was fixed at a value of 5 × 10^–7^ MPa^–1^, taken from the study of the lipid DPPC by Duncan and Larson (2008). The twin range cutoffs were set at 0.8 nm for DGDG simulations, while PE monolayers were simulated with both 0.8 and 1.6 to test whether a shorter value would properly represent the P–N interactions within the PE head group. The dynamic behavior of the monolayers was found to be identical in both cases.

The original configurations were constructed in stages, using the packmol program (Martínez et al., 2009). In the DGDG simulations, the water slab contained 40,000 molecules in an approximate volume of 18 × 18 × 6 nm. For PE, the number was 60,000. The two surfactant monolayers decorating the slab were then constructed by semi-random insertions of DGDG (276 molecules per monolayer) or PE (280 molecules per monolayer), with two restrictions: (1) That no two atoms be closer than 2 Å from one another, and (2) That the head group atoms were always placed closer to the water slab than the tail group atoms.

These configurations were energy minimized using the steepest descents algorithm, to allow the bonds and angles to relax closer to the constraints in the topology file. Next, the pressure and temperature coupling were activated, and the system equilibrated for 4 ns to generate the input coordinates for the data collection runs. Velocities were generated to fit a Maxwell Boltzmann distribution at 298 K, with the random seed changed in each simulation.

### Pore Area Calculation

**FIGURE 2:**
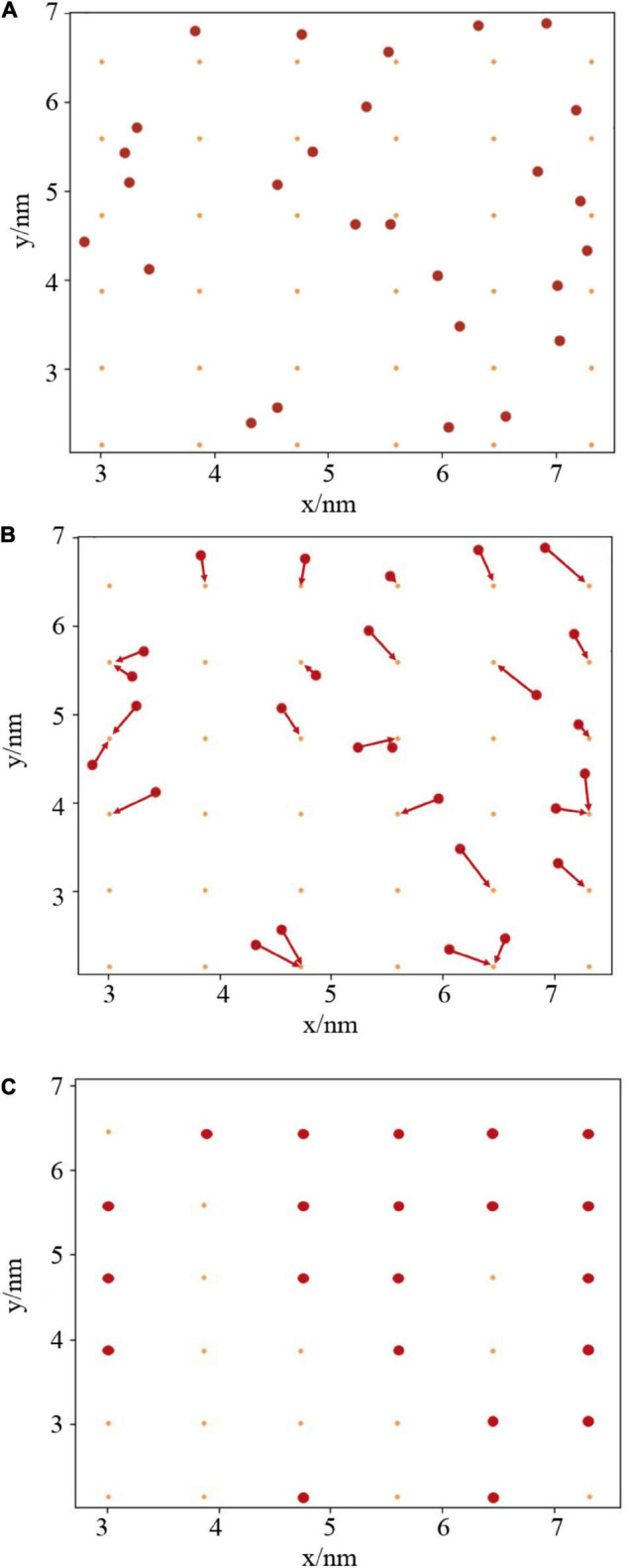
**(A)** Two dimensional projection of lipid atoms and grid points. **(B)** Rounding of atoms to nearest grid points. **(C)** Calculation of total pore area as the proportion of unoccupied (orange) to occupied (red) grid points.

To understand the stability and integrity of the monolayers, a script was written to chart the exposed area of water, in two dimensions, as a function of time. Taking inspiration from the works of Gonzalez et al. (2015) and Wang and Frenkel (2005), the cavities in the monolayer were determined by comparison of the head group positions with a two-dimensional grid, as shown in Figure 2. Briefly, the algorithm works as follows:

**Step one:** An appropriate atom is chosen to represent the head group position of each lipid. In this case, phosphorus and nitrogen were chosen for PE, and all oxygens present within the galactosyl groups for DGDG. The *x* and *y* coordinates of these *N* atoms are then read in using the mdtraj python module (McGibbon et al., 2015).

**Step two:** create an *M × M* grid, slightly offset from the box walls, where *M*^2^ is approximately equal to the number of atoms read in above (panel **A**). Generate a corresponding *M × M* counting matrix, populated with zeros.

**Step three:** For each time step, round the atomic coordinates to the nearest grid point (panel **B**). This is achieved by dividing every point by the grid spacing, and then converting to an integer. If more than one atom is present at each grid point, add one to the corresponding element of the counting matrix. The pore area at each timestep is then calculated as proportion of zeros in the counting matrix (panel **C**).

The advantage of this algorithm over that of Gonzalez, or Voronoi tessellation methods like those used by Wang and Frenkel (2005) to study monolayer rupture, is that at no point are the grid positions directly compared with atom positions, nor are any magnitudes calculated. This grants significant speed boosts when analyzing long trajectories since both of those procedures scale much more strongly with *M* and *N* than division of a vector by a scalar.

### Surface Tension Calculation

Unless otherwise stated, the value of γ_*water*_ used in this study was 40 mNm^–1^, as opposed to the real value of 72 mNm^–1^. This is based on underpredictions of γ_*water*_ by the TIP4P-2005 water model, when simulated with twin cutoff values of 0.8 nm (Javanainen et al., 2018). Surface tension has been calculated in two ways: The pressure tensor method for the main analysis, and the capillary wave method as a sanity check.

*The pressure tensor (PT) method* (Vega and de Miguel, 2007) involves subtracting the normal elements, *P*_*x*_ and *P*_*y*_, from the perpendicular element, *P*_*z*_, of the pressure tensor as a function of time.


(2) $$\gamma_{P\,T}=\frac{1}{n}\,h_{z}\,\left[P_{z}-\frac{1}{2}\,\left(P_{x}+P_{y}\right)\right]$$

where *h*_*z*_ is the vertical height of the simulation box, and n is the number of interfaces present (in this case 2). In simulations when the surface tension is expected to be constant, or to approach equilibrium as time proceeds, one can calculate γ*_*PT*_* as a cumulative sum.

*The capillary wave (CW) method* relies on the intrinsic relationship between the surface tension and the width of the interface (Nickerson et al., 2013). It is useful here as it allows independent determination of the tensions of each monolayer separately. It requires the calculation of two length scales which have dubious physical meaning: the interfacial variance, or fluctuation, and the molecular diameter.


(3) $$\gamma_{C\,W}=\frac{k_{B}\,T}{2\,\pi \,\sigma^{2}}\,\ln\left(\frac{h_{z}}{l}\right),$$

where σ is the interfacial variance, and *l* is the molecular diameter. Here, we have calculated σ using the lateral density of the simulation box, by fitting an error function. Molecular diameter is calculated as the mean of the water Van der Waals diameter (2.8 Å) and the diameter of gyration of the lipid head groups,


(4) l=2⁢hx⁢y2π⁢nl⁢i⁢p⁢i⁢d⁢s

where *h*_*xy*_ is the simulation box width. Therefore, *l* varies as a function of time. γ_*CW*_ is calculated in 500 ps chunks, by dividing the *z* dimension of the simulation box into 300 slices.

**FIGURE 3:**
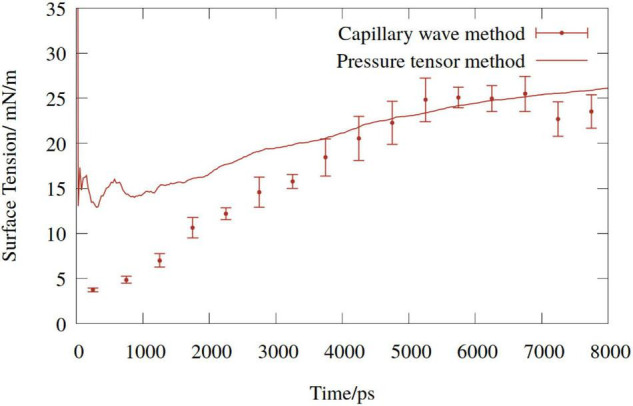
Time dependent surface tension of digalactosyldiacylglycerol (DGDG) monolayers (mean of upper and lower) pulled at a pressure of −1.5 MPa. Solid line represents the cumulative average of the pressure tensor estimate, points represent the capillary wave method.

A comparison of the time dependent values of γ_*CW*_ with the cumulative mean of γ*_*PT*_* for one of the DGDG simulations conducted is shown in Figure 3. Given that σ in Equation (3) is a function of molecular coordinates, γ_*CW*_ begins much lower than γ*_*PT*_*, and increases over the first few nanoseconds, proportionally with the pulling rate. By contrast, γ*_*PT*_* is dependent only on the atomic forces, and so adopts larger values at earlier times.

The two methods converge, and a reasonable agreement is reached at around 4 ns. At no point in any simulation did the γ_*CW*_ estimates of the top and bottom monolayers deviate by more than the variance within one 500 ps bin, or show dissimilar trends. Therefore, the pressure tensor value was considered an accurate representation of the “mean” monolayer behavior, and so was chosen for the analysis.

## Results

**FIGURE 4:**
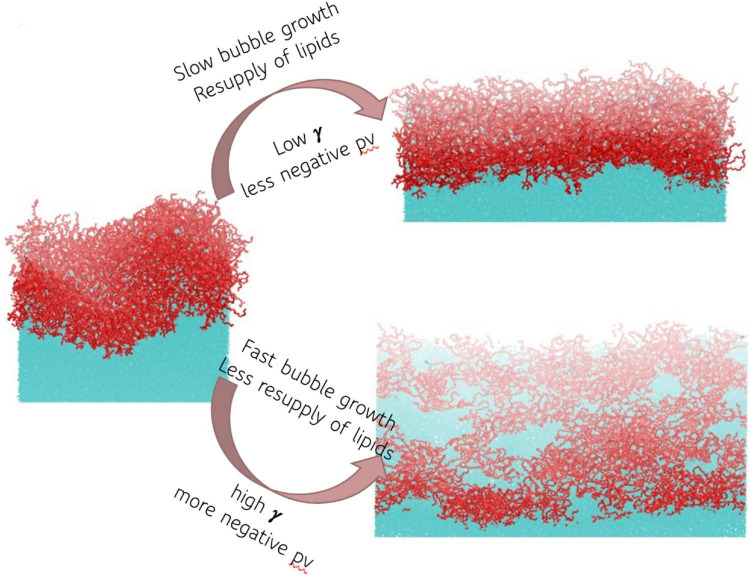
Schematic representation of the effect of pressure and bubble growth rate on monolayer structure, and hence surface tension γ. Red atoms are digalactosyldiacylglycerol (DGDG), cyan are water.

In the current study, we applied four biologically relevant negative pressures to the glycolipid digalactosyldiacylglycerol (DGDG), between –0.5 and –3.5 MPa inclusive. DGDG was identified in the sap in a recent landmark publication (Schenk et al., 2021) using electrospray ionization mass spectrometry to characterize the lipid composition of xylem sap of seven angiosperm species. In all but the lowest tension case (−0.5 MPa) rupture of the monolayer was observed, exposing the water surface below. Snapshots of one DGDG interface before and after pulling at two example pressures is shown in Figure 4. Note the square nature of the interface, and the radically different areas of water exposed in the two right hand schematics.

The phospholipid Phosphatidyl ethanolamine (PE), another lipid present in sap (Schenk et al., 2021), was also investigated. As we will see in the following section, its monolayers were found to be completely resistant to pulling down to pressures of −5.5 MPa, which exceeds the range of tensions commonly found in plants.

### Digalactosyldiacylglycerol Monolayer Rupture and Pore Formation

Both experimental and computational (Baoukina et al., 2007; Javanainen et al., 2018), studies have shown that lipid monolayers transition through several phases, depending on the area available: from liquid-condensed *L*_*c*_, to liquid expanded *L*_*e*_, to a ruptured network of pores surrounded by otherwise intact regions of *L*_*e*_ [for schematic representations of the phases we recommend the publications of Baoukina et al. (2007) and Duncan and Larson (2008)]. Note that unlike previous literature, the monolayers here are being actively pulled to mimic negative xylem pressures, rather than simply allowed to relax to a reduced *positive* pressure.

**FIGURE 5:**
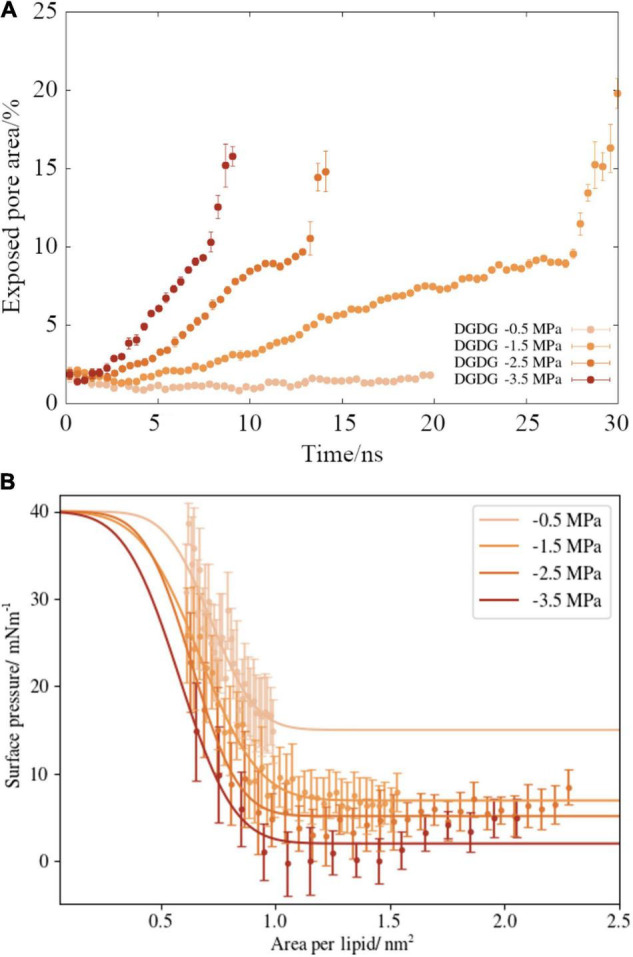
**(A)** Time dependent ruptured pore area for digalactosyldiacylglycerol DGDG monolayers, as a function of (external) negative pressure. Values calculated after the water slab began to rupture have been removed for clarity. **(B)** Surface pressure area isotherms for the same monolayer pulling simulations (points), alongside best fits to Equation (6) (lines). Error bars represent one standard deviation of the averaged data.

A script was written to determine the area of exposed water in every frame of the MD trajectory (as described in section “Materials and Methods”). This analysis was applied to the lipid DGDG and the results are presented in Figure 5A. We can see that a more negative pressure causes a faster increase in the pore area within the lipid monolayer during the pulling. The monolayers experiencing the most modest pressure (−0.5 MPa) maintained a sufficient lateral force that the water slab was stopped from embolizing (boiling). Interestingly, at the three pressures during which rupture was observed, the nanobubble monolayers lost integrity at the same pore coverage: approximately 10% of the surface area. Once that threshold was reached, runaway growth took hold, signifying embolism would have occurred at that point within a xylem vessel.

### Dynamic Surface Tension and Surface Pressure

In the initial configuration of the DGDG simulations, the monolayer is highly doped and buckles, or warps, slightly to fit all of the lipids in. Such a configuration is a consequence of the fact that the head group area is significantly larger than the cross-sectional area of the tail (Israelachvili, 2011). Concomitantly, the starting surface tension, at 5.3 mNm^–1^, is extremely low (see Figure 3). By contrast, PE is more capable of lamellar packing at this length scale, due to the head group being closer in area to the footprint of the tail.

In the literature, surface *pressure*, Π, is the preferred metric for quantifying interfacial properties. We have chosen to express our results in this format. Conversion of surface tension to surface pressure was achieved by subtraction of the calculated surface tension from the water surface tension determined by the same method, for the same model:


(5) $$\Pi \,\left(A\right)=\gamma_{w\,a\,t\,e\,r}-\gamma_{i\,n\,t\,e\,r\,f\,a\,c\,e}\,\left(A\right)$$

Upon application of negative pressure coupling, the interface is stretched at rates proportional to the applied tension. Therefore, the ability of the lipids to maintain order is disrupted and, as shown in Figures 4, 5A, the monolayers lose structural integrity. We present the surface pressure area isotherms (named by analogy with pressure volume isotherms of gases) of DGDG in Figure 5B, which shows that Π(*A*) begins to decrease toward zero (the point at which the interface behaves as if no lipids were present) rapidly in all four simulations. Indeed, for the −3.5 MPa pressure, the observed surface pressure reaches zero in the region 0.9–1.5 lipids nm^–2^.

Time averaged quantities, such as Π, fluctuate significantly during molecular dynamics simulations, which can be a problem when extrapolating the data to predict properties of larger systems. In order to present the CNT free energy as a smooth function of radius in section “Discussion,” the calculated pressure area isotherms should be parameterized as simple, monotonically varying functions. Ideally, a physically realistic representation of Π*(A*) will plateau at low *A*, where the monolayer is stable. It will then transition to a much lower value at high *A*, as the monolayer ruptures. The error function was identified as containing these features, and was thus selected for this purpose:


(6) $$\Pi \left(A\right)=\Pi_{0}-\frac{\Pi_{r\,u\,p\,t\,u\,r\,e}}{2}\cdot (1+\text{erf}\left(b\left(A-c\right)\right)$$

Here Π_0_ is the surface pressure before the transition, Π_*rupture*_ is the surface pressure *difference* between the ruptured and unruptured configurations. During the fitting of Equation (6) to the data, the fit parameter *b* and *c* were introduced to determine the shape and center of the transition, respectively. We chose to fix the value of Π_0_ to be γ_*water*_—ε, where ε is the mean absolute error of the surface pressure for each external pressure (i.e., the mean size of the bars in Figure 5B). The fitting was carried out using the curve_fit function in python, with the following bounds: 0.1 < *b* < 5, 0.5 < *c* < 1.2*nm*^−2^, and 2 < Π_*rupture*_ < 20 mNm^−1^.

The best-fit curves are presented alongside the appropriate data points in Figure 5B. The influence of external pressure on surface pressure can be seen by the fact that Π_*rupture*_, the width of the transition, increases monotonically as *p* becomes more negative.

Calculating the corrected γ_*interface*_(*A*) values using Equation (5) produces a “true” surface tension range accessed by the DGDG simulations of between 36 and 73 mNm^–1^ inclusive of all pressures. We note that the range of corrected γ_*interface*_ values exhibited at −1.5 MPa pressure is 46–67 mNm^–1^, which is almost identical to the bulk xylem sap surface tensions experimentally measured by Losso et al. (2017) from the gynosperm species *Picea abies* and *Pinus mugo*. Bulk xylem measurements in angiosperms (Christensen-Dalsgaard et al., 2011) have produced slightly larger values (55–70 mNm^–1^), although it should be noted that bulk sap will have a lower average lipid concentration than a nanobubbles internal surface.

There is a small increase in the calculated surface pressure at 0.8 nm^–2^ in the −0.5 MPa case (Figure 5B). Similar “activation barriers” have been observed in MD simulations of monolayers before (Javanainen et al., 2018), and are indicative of the transition from a mostly *L*_*c*_ phase structure to a mostly *L*_*e*_. The presence of such a barrier in our data gives confidence we have accurately modeled the phase characteristics of the system. It also suggests that monolayers experiencing less negative pressures behave more conventionally than those under more extreme tensions.

### Stability of Phosphatidyl Ethanolamine Monolayers

Three monolayer pulling simulations were conducted using the phospholipid PE, each beginning from the coordinates shown in Figure 1D, at external pressures −1.5, −3.5, and −5.5 MPa. We found PE monolayers to be highly resistant to pulling: Even tensions exceeding those commonly observed in xylem (−5.5 MPa) (Lintunen et al., 2013; Losso et al., 2017) were not able to produce significant changes in monolayer area on the timescale of the simulation (the lateral box size increased by less than a 2% over 22.5 ns). Similarly, while the calculated surface tensions, γ*_*PT*_*, were different in each simulation, the values fluctuated much less, as a function of time, relative to the glycolipid monolayers.

By exhibiting both lateral stability and numerically stable surface tensions, it follows that PE monolayers occupy a single point in surface pressure-area space, rather than the sigmoidal function we used to represent the glycolipid DGDG in Figure 5B. We therefore consider the behaviors of these two lipids to be different in kind, rather than degree (at least within the pressure range studied). The implications of this for mixed monolayers will be discussed in the next section. For the time being, we can say that the surfactant (or surfactants) present on the surface of a nanobubble will radically alter its fate within the xylem.

The two-dimensional structure of the PE monolayer is also distinct from DGDG: The head groups of PE do not organize themselves across the interface at a uniform density. Instead, they adopt a two-dimensional structure that resembles filaments when viewed from above. Much of the surface water is not bonded directly to a head group, leading to an equilibrium pore area of ∼23%. The benefit of such a configuration appears to be to maximize the contact between the P and P/N atoms of adjacent head molecules, which may account for the lateral stability of PE.

Comparison of the two lipid head groups shows that they have a similar number of neighbors, as determined by calculating their coordination numbers (2.35 for PE vs. 2.59 for DGDG). However, the first solvation shell of PE is significantly smaller than that of DGDG (6 vs. 15 Å), implying that the phospholipid bonds more closely and strongly to itself. We show a snapshot of the configuration, alongside the relevant radial distribution functions of Phorphorus and Nitrogen in Supplementary Figure 5.

## Discussion

While this study and our results belong to computational or soft matter physics, they have important implications for nanobubble stability under tension and thus, for plant physiology. Therefore, we would like to expand and elaborate on some of the connections.

### Pressure-Area Isotherms

Returning to Figure 5B, we can see that the four sets of points do not overlap significantly in pressure/area space. Instead, more negative external pressures lead to lower surface pressure at a given bubble size. We can therefore infer that the lipid structure has less time to re-organize in response to the interface expanding at more negative pressures, as the area increases faster than the lateral diffusion of lipids can compensate for.

What these results show is that there is a negative feedback loop present at the surface: The more rapid the expansion of the monolayer, the larger the increase in surface tension. Paradoxically, more negative pressures will lead to more stable bubble surfaces with respect to embolism, because the energetic cost of continuing to grow the surface is much higher. One can make an analogy here to a non-Newtonian fluid: a slow deformation will be met with minimal resistance, whereas the application of a great force will cause an instant vitrification in kind. We therefore propose that this effect is the crucial factor determining the fate of each bubble. By extension, if trees are able to regulate lipid concentration such that a certain bubble size is favored, there may be the means by which trees have evolved to tolerate some bubbles entering the xylem, while suppressing their ability to embolize.

### Classical Nucleation Theory

We have just shown that the surface of an expanding nanobubble will act in such a way as to counteract that expansion. However, the Gibbs free energy of a nucleation process is also a function of the volume of the new phase. Is the surface tension increase sufficient to counteract the mechanical work extracted, *pv*, by increasing the gas volume per bubble? To answer this, we have calculated the formation free energy of a hypothetical DGDG covered nanobubble using a CNT approach:


(7) $$g\,\left(r\right)=4\,\pi \,r^{2}\,\frac{\gamma_{i\,n\,t\,e\,r\,f\,a\,c\,e}\,\left(r\right)}{1+2\,\delta /r}+p\,v\,\left(r\right)$$

Here *r* is the bubble radius, *v* its volume and δ the Tolman length. *p* is the pressure difference between the internal bubble pressure and the external negative pressure. The size dependent surface tensions, γ_*interface*_(*r*), were produced from the error function parameterizations presented in section “ Materials and Methods.” To convert γ from a function of area per lipid into a function of bubble radius we have assumed that, at each pressure, the bubbles will have a unique number of lipids, *n*_*lipids*_, coating their inner surface. Here we have chosen *n*_*lipids*_ = 30,000 for all four pressures. We present in the Supplementary Information two further sets of *g(r)* curves, assuming *n*_*lipids*_ is pressure dependent. We find that the general shape of the curves remains similar.

A note on the Tolman length: δ is an abstract model parameter with no concrete experimental counterpart, and a wide range of values have been proposed in studies modeling nanobubbles, or water under negative pressure: They range from –0.047 nm (Azouzi et al., 2013; Yasui et al., 2016) to 17 nm (Manning, 2020). Here we have chosen 0.2 nm, the value used by Menzl et al. (2016) to simulate the cavitation of pure water.

**FIGURE 6:**
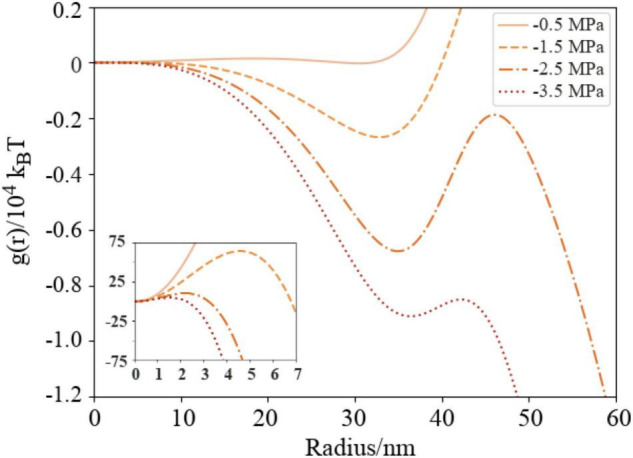
Naïve Gibbs free energy surfaces (Equation 7) for DGDG as a function of external (negative) pressures in a nanobubble with varying radius, presented in units of 10^4^ k_*B*_T. *P*_*external*_ = -0.5 MPa (solid line), −1.5 MPa (dashed), −2.5 (dash-dotted) and −3.5 (dotted). Insert figure shows cavitation free energy barriers in the region *r* = 0–7 nm in units of *k_*B*_T*. Color scheme is the same as Figure 5.

The formation free energy curves calculated for DGDG coated bubbles at four negative pressures are shown in Figure 6. All exhibit a two barrier “kinetic trap” type dependence on bubble radius [visible as local minima in *g(r)*]. The first potential barrier, which can be seen in the insert figure, is the barrier to homogeneous cavitation, i.e., formation of a small volume of vacuum with a surface tension close to zero (Abascal et al., 2013). The second barrier is the barrier to embolism. In all cases, the metastable radius is approximately 35 nm: a bubble of this size lies at a local minimum of Gibbs free energy where it is thermodynamically unstable relative to a fully embolized vessel, but kinetically trapped from achieving embolism. A separate calculation of surface entropy across this radius range is presented in Supplementary Material.

Metastability in nanobubbles is not a novel concept: It was proposed recently by Yarom and Marmur (2015), who hypothesized that a bubble of insoluble gas can become kinetically stabilized when its size is equal to the critical radius. Such a mechanism is similar to that experienced by small aerosol particles, and described by the so-called Kohler theory (Köhler, 1936). By contrast, we are proposing here a nanobubble containing soluble gas achieving metastability at a potential energy minimum, rather than maximum.

A literature survey was conducted to ascertain whether a size distribution centered at 35 nm was physically realistic given the air seeding model of bubble formation. Our results were found to compare very favorably with Boutilier et al. (2014), who filtered solid particulates through a pine branch, extracting a distribution centered at a radius of 40 nm. By contrast, Choat et al. (2003) observed that only nanoparticles between 5 and 20 nm were able to pass through a pit membrane. This may not preclude the existence of larger bubbles, however, as they could adopt an elongated shape during seeding, with a small cross-sectional area.

The −0.5 and −1.5 MPa curves contain enormous barriers to embolism, meaning that were a distribution of air-seeded bubbles to be subject to either energy landscape, they would *never embolize*. Instead, each bubble would either expand or contract from its original size until it reached the local minimum. Any further attempt to expand would be met with significant resistance, even if lipids could be resourced from nearby to stave off monolayer rupture.

Conversely, any reduction in radius from the local minimum also destabilizes the bubbles: they approach the activation barrier for cavitation (the peaks at ∼5 nm in the inset Figure 6) from the other side. Physically, one would expect a concomitant increase in internal pressure to occur, and hence an outward force acting to restore the radius to its metastable value, reducing the likelihood of sub 10 nm bubbles. The existence of these barriers support the conclusions of the recent publication by Vehmas and Makkonen (2021), which suggests that bubble dissolution becomes unlikely at small radii as the internal pressures increase significantly.

As Schenk et al. (2017) have pointed out, surface area to volume ratios dictate that fragmentation into several smaller bubbles is energetically favored over expansion, assuming curvature effects can be discounted. We therefore propose that, at xylem sap tensions between 0 and at least –1.5 MPa, the dynamic behavior of nanobubbles is characterized by a mechanism of continuous expansion, fragmentation, and recycling into smaller bubbles. Mechanistically, what occurs may resemble the transitions between nanobubbles and liposomes observed by Koshiyama and Wada (2016), where the coating monolayers buckle and fold inward, creating multiple internal surfaces inside the bubble. Clearly, further work is needed to more fully validate the existence of this process on a microscopic level.

For the −2.5 and −3.5 MPa cases, the peaks of the barriers to embolism are below the 0 free energy level. Physically speaking, this means that the critical bubble will be more stable than the same amount of gas dissolved within the liquid. Therefore, bubbles should have sufficient thermal energy to embolize at these pressures, assuming the energy is not immediately dissipated into the surrounding tissue, or tapped by the tree in some other way.

A similar phenomenon has been observed in bimolecular chemical reactions (Glowacki et al., 2011), whereby products retain vibrational or thermal energy for extended periods, without dissipation, allowing them to undergo further transformations. Alternatively, if the residual Gibbs free energy is quickly drained, the bubbles will adopt the local minimum radius and remain metastable, like those experiencing lower tensions. We therefore consider the rate of energy dissipation directly after air seeding occurs to be a key parameter in determining the stability of nanobubbles in the xylem vessels.

Given that tension within the xylem sap increases from the roots to the leaves, one could propose that a bubble moving upwards within a xylem vessel will be subject to each of these energy surfaces in turn. In such a situation, the mechanical work of formation becomes more negative, but this is offset by the interfacial tension of the bubble increasing. Furthermore, nanobubbles will cross multiple pit membranes on their journey upwards, which could change their coating.

An increase in the surface tension of a nanobubble, but no increase in size, may allow for gradually higher internal gas pressures to be sustained, potentially as high as those observed by Ohgaki et al. (2010). The Laplace pressure, defined as


(8) $$p_{L\,a\,p\,l\,a\,c\,e}=\frac{2\,\gamma \,\left(r\right)}{r}$$

allows us to estimate the equilibrium vapor pressure within a spherical bubble. A few instructive examples: Inside a 35 nm bubble with a γ*(r)* of 47 mNm^–1^ (Π = 25 mNm^–1^), *P*_*Laplace*_ is +2.7 MPa, rising to +4.1 MPa for a γ*(r)* of 72 mNm^–1^ (a plot of estimated *P_*Laplace*_* against *P*_*external*_ is provided in Supplementary Figure 3). These values suggest that nanobubbles under high tension can transport more gas by molar quantity than those under less negative external pressures, even if they are the same size. We note that the critical pressure of N_2_ is 3.4 MPa (Lemmon et al., 2021), meaning that in extreme cases the air present in nanobubbles may in fact condense into a supercritical state. Additionally, CO_2_ and O_2_, which are both present in xylem sap, are believed to be moderately surface active at high pressures, and so should migrate to the interface whenever any pores in the monolayer form. The precise interplay of these phenomena, and their effects on bubble stability, remains elusive.

### Possible Effects of Mixed Monolayers

It is highly likely that the surface coating of each nanobubble within xylem sap contains multiple different surfactants, at varying concentrations, or even small proteins such as saponins (Christensen-Dalsgaard et al., 2011) which are known to interact with lipid bilayers in plant cell walls (Lin and Wang, 2010). Indeed, given that the lipids present at pit membranes mostly originate from cells that have died, it stands to reason that their phase behavior echoes, to an extent, that of the membranes from which they came.

Combining lipids which are highly resistant to pulling with those more easily ruptured could potentially allow the organism to tune the pressure range (for which there is a huge variability from species to species; Choat et al., 2012) within which bubbles can survive. Our results hint at such a variability: Of the two lipids investigated herein, only one (DGDG) accommodated any expansion of its monolayers at the pressures investigated. By contrast, PE was shown to have remarkable resilience within the range of biologically relevant tensions (we note that PE was a minor constituent, relative to DGDG, in all seven angiosperms investigated by Schenk et al., 2021). The behavior of other phospholipids under tension, or indeed mixed monolayers of glyco- and phospholipids, remains elusive.

## Conclusion

Here we have used state of the art molecular dynamics methods to directly calculate the surface tension of two biologically relevant lipids that can be expected to coat nanobubbles at a range of negative pressures found in trees. The values calculated for the glycolipid DGDG are in line with previous measurements of bulk xylem sap surface tension. We discover that lipid monolayers are less capable of rearranging at more negative pressures, destabilizing the internal surface but stabilizing the nanobubble with respect to runaway growth. CNT predicts that, at external pressures between 0 and –1.5 MPa, this effect is sufficient to avoid embolism altogether. At more negative pressures, embolism becomes increasingly likely, depending on the rate of free energy dissipation. We propose a mechanism wherein xylem nanobubbles repeatedly expand and collapse into smaller bubbles, recycling their surface lipids in the process. These novel results reconcile the existence of nanobubbles in xylem sap with the cohesion-tension theory of water transport in plants.

To our knowledge, this is the first time a dynamic surface tension has been shown to be influenced by pulling rate (i.e., varying external tensions) rather than by more common hysteresis effects which depend on whether the interface is expanding or compressing. We are also unaware of any direct comparisons of surface energy expended to the mechanical work gained by boiling in the context of air seeding, rather than homogeneous cavitation. Further work will investigate the impact of significantly elevated Laplace pressures within the bubble on interfacial tensions. In the future, we aim to explore the phenomenon of lipid resupply to an expanding interface, in the form of micelles or other self-assembled structures, and the rate at which it proceeds.

## Data Availability Statement

The raw data supporting the conclusions of this article will be made available by the authors, without undue reservation.

## Author Contributions

SI conducted the simulations and analysis, under the supervision of HV and TV. SI wrote the majority of the manuscript. YS and TH were part of the same academy of Finland funding consortium, and wrote small parts of the manuscript during revisions. Along with AL, they shaped the project through multiple interdisciplinary meetings throughout 2019 and 2020. All authors contributed to the article and approved the submitted version.

## Conflict of Interest

The authors declare that the research was conducted in the absence of any commercial or financial relationships that could be construed as a potential conflict of interest.

## Publisher’s Note

All claims expressed in this article are solely those of the authors and do not necessarily represent those of their affiliated organizations, or those of the publisher, the editors and the reviewers. Any product that may be evaluated in this article, or claim that may be made by its manufacturer, is not guaranteed or endorsed by the publisher.

## Footnotes

1
https://limonada.univ-reims.fr/

2
https://atb.uq.edu.au/
