# Supplementary material for: Dynamic Surface Tension Enhances the Stability of Nanobubbles in Xylem Sap
Source: Front Plant Sci. 2021 Dec 16;12:732701. doi: 10.3389/fpls.2021.732701 (PMC8716698; doi:10.3389/fpls.2021.732701)
Supplement: Supplementary file 1 [file Data_Sheet_1.docx]

Supplementary Information for: Dynamic Surface Tension Enhances the Stability of Nanobubbles in Xylem Sap

Stephen Ingram^1^, Yann Salmon^1,2^, Anna Lintunen^1,2^, Teemu Hölttä^2^, Timo Vesala^1,2,3^, Hanna Vehkamäki^1^

^1^ Institute for Atmospheric and Earth System Research, University of Helsinki, 00560 Helsinki, Finland

^2^ Institute for Atmospheric and Earth System Research/Forest Sciences, University of Helsinki, 00790 Helsinki, Finland

^3^ Laboratory of Ecosystem-Atmospheric Interactions of Forest - Mire Complexes, Yugra State University, 628012, Khanty-Mansiysk, Russia

*Physical meaning of negative pressure and negative surface pressure*

We note that in the vast majority of published isothermal-isobaric ensemble simulations the external pressure applied is positive. Mathematically speaking, this means that fluctuations in the outward force exerted by the molecules against the simulation box are damped and the density approaches, and then stays near its equilibrium value. A *negative* pressure in this context means that the box walls will move in the same direction as, rather than against, the force exerted by the molecules, as determined by the *xx* and *yy* elements of the pressure tensor: The effect is that the simulation will act to continuously reduced density over time. More simply; the monolayer is pulled apart.

Negative surface pressure values have been observed in molecular dynamics (MD) simulations before, for example by Baoukina et. al.^1^, but they have also been described by Javanainen et al.^2^ as unphysical.

*Effect of the number of lipids on g(r)*

When choosing values for *n_lipids_* in the main text, we decided that it should remain constant. Here we consider two further situations:

Firstly, where resupply of new lipids to the interface will become slower, relative to the timescale of the expansion, as the tension increases. In Figure 5a in the main text, both increases in pressure from –3.5 to -2.5 MPa, and from -2.5 to -1.5 MPa roughly doubled the time to rupture in the simulation. Therefore, assuming a constant diffusion rate over time, the extra lipids that will coat a bubble during its expansion will approximately halve for every 1 MPa reduction in pressure (*p*). This adds to an assumed 8000 ‘starting’ lipids present immediately after air seeding.

| **p/MPa** | **n_lipids_** |
| --- | --- |
| -0.5 | 88000 |
| -1.5 | 48000 |
| -2.5 | 28000 |
| -3.5 | 18000 |


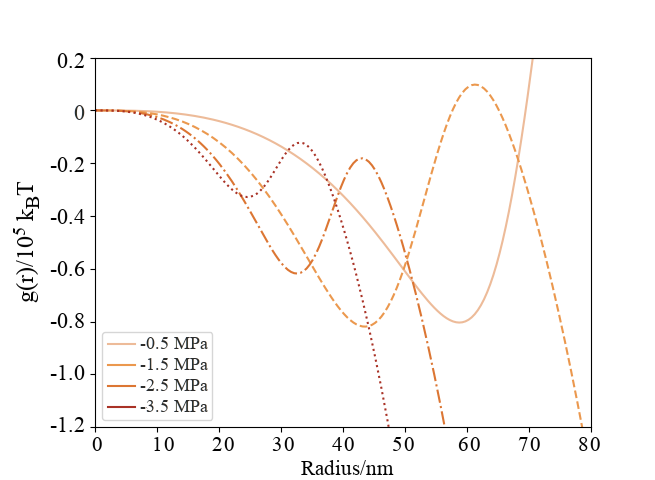


**Figure S1:** Naïve potential energy surfaces (eq. 4) for DGDG, presented in units of 10^5^ k_B_T, assuming n_lipids_ are distributed according to Table 1.

We see that the metastable radius decreases significantly, from 62 to 23 nm, as the pressure becomes more negative. Like Figure 6, the barrier to embolism exceeds 0 free energy for the –0.5 and –1.5 MPa curves, and does not for the other two.

Secondly, we propose the opposite trend, namely that *n_lipids_* increases as *p* becomes more negative. A possible justification for this is that it represents the journey a single bubble will take as it rises through the tree. Over time, the cumulative number of lipids that could have migrated to the interface will increase.

| **p/MPa** | **n_lipids_** |
| --- | --- |
| -0.5 | 18000 |
| -1.5 | 28000 |
| -2.5 | 48000 |
| -3.5 | 88000 |


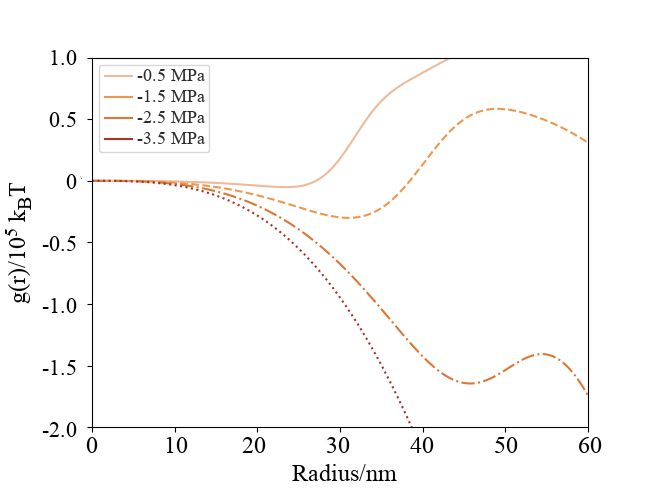


**Figure S2:** Naïve potential energy surfaces (eq. 4) for DGDG, presented in units of k_B_T, assuming n_lipids_ are distributed according to Table 2.

In this case, the metastable radius increases with *n_lipids_*. As above, the barriers to embolism exceed 0 k_B_T for pressures less negative than –2 MPa. Uniquely, there is no second barrier in the –3.5 MPa case, meaning bubbles would immediately embolise if they were exposed to this pressure in the presence of a very large number of lipids.

*Internal Laplace pressure versus external pressure*

As stated in the main text, the Laplace pressure of a small bubble is determined by equation 5. By assuming a metastable radius of 35 nm (Figure 6), and surface tensions at the precise center of the error function transition (𝛾_water_ - Π_rupture_/2, Figure 5b), it is possible to calculate *P_Laplace_* as a function of P_external_, as shown in Figure S3, below.


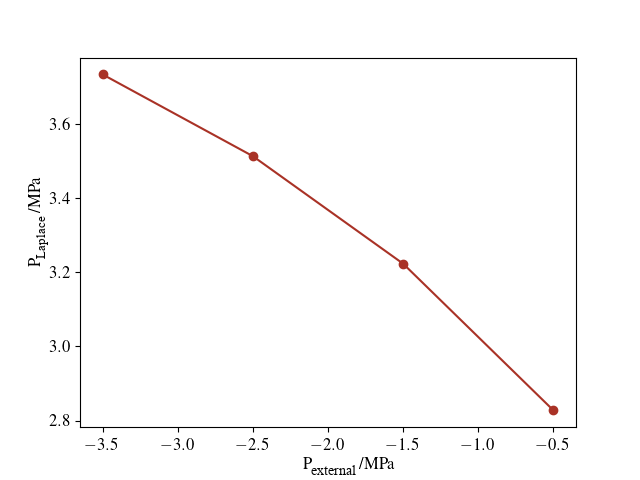


**Figure S3:** Comparison of the internal Laplace pressure within a 35 nm bubble, and the applied (negative) pressure. Surface tensions, 𝛾(r), are taken as the center of the S-curves in Figure 5b.

*Bubble surface entropy*

The free energies calculated in section III are more accurately described as Gibbs free energies, as they take place in an isothermal-isobaric ensemble (NpT), hence they are denoted by the symbol *g*. While the entropic contribution of the new phase to the free energy is already accounted for within equation 2, it can be determined independently.


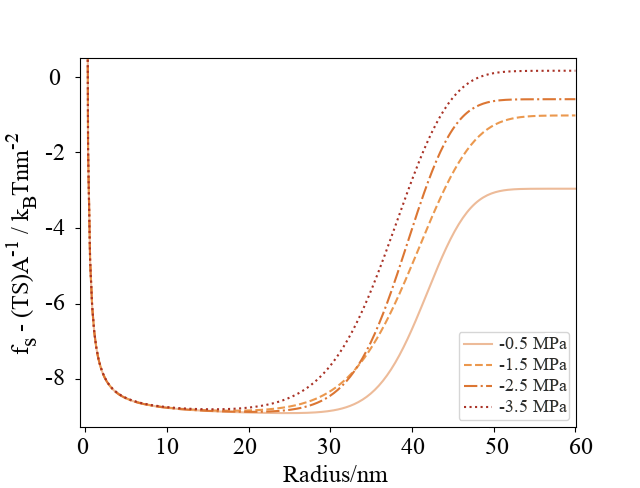


**Figure S4:** Difference between bubble surface free energy and bubble entropy per unit area (dependence reproduced from ref. 3).

Entropy calculations for a system very similar to this one (cavitation in TIP4P-2005 water at close to 0 pressure) have been done previously by Menzl et. al.^3^. Here we take their results and compare them with the *surface free energy* of the bubble, using the equation:

$$f_{s}\left( r \right)=\frac{\gamma\left( r \right)}{1-\frac{2\sigma}{r}}-\frac{0.0972}{4{\pi r}^{2}}$$

also from ref. 3. Where all terms are as defined in the main text, and *f_s_* is expressed in units of *k_B_T*. Subtracting the value of the entropy gained through nucleation, per unit area, from *f_s_*, produces the curves shown in figure S4. The entropy function is a linear relationship between (TS)A^-1^ and r^-1^

$$\frac{TS}{A}=2.5\cdot(3.6-\frac{1}{r})$$

In units of *k_B_T* nm^-2^. The two variables were fit to the red points presented in figure 4 of the Menzl paper^3^. It can be seen from figure S4 that the two contributions are approximately balanced. Cavitation releases ~9 kT per square nanometer in entropy, between 5 and 9 kT of which is consumed as the monolayer ruptures. This is another reason why nanobubbles are metastable: Excluding the contribution of the mechanical work, *pv*, the interface of a large bubble with a high *𝛾* is only slightly more stable than if no interface, and hence no bubble, was present. Note that this calculation does not account for the entropy that would be released during monolayer rupture, which may be substantial.^4^

*The structure of a Phospholipid monolayer*


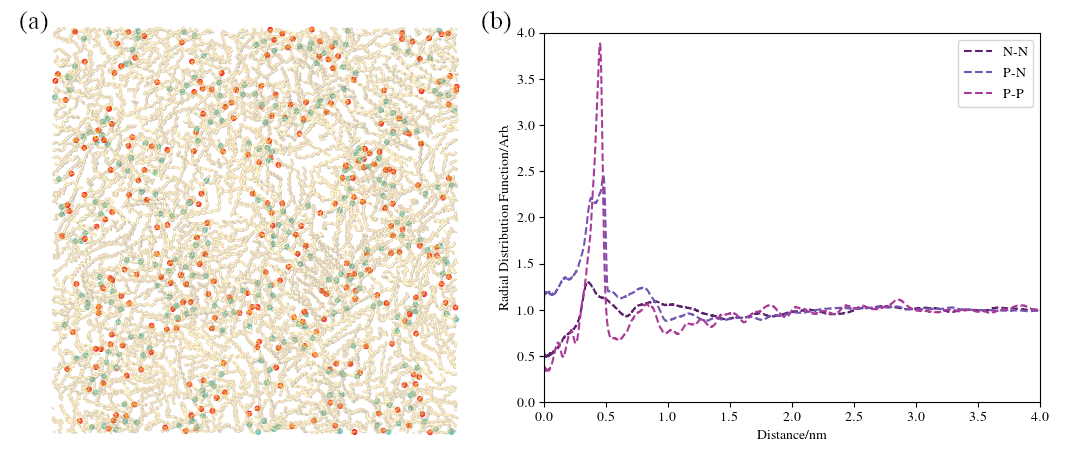


**Figure S5:** (a) Structure of a Phosphatidyl ethanolamine (PE) monolayer, averaged over 22.5 ns, as viewed in the *xy* plane (green atoms are phosphorus, red are nitrogen, all others are orange). (b) The associated radial distirbution functions for head group atoms.

As described in the main text, the PE monolayer system was found not to fluctuate significantly during 22.5 ns pulling at -5.5 MPa. We present plots of the two-dimensional P-P and P-N Radial Distribution Functions (RDFs) in Figure S6, panel(b), showing that they both associate at a bond distance of 4.5 Å. This is nearly half the length of the gyration radius of each head group (9.3 Å), but is consistent with RDF peaks previously calculated for bilayers comprised of the phospholipid dipalmotoylphosphatidylcholine (DPPC).^5^ The phosphorus – phosphorus correlation is significantly stronger than the other pairs.

**References**

(1) Baoukina, S.; Monticelli, L.; Marrink, S. J.; Tieleman, D. P. Pressure-Area Isotherm of a Lipid Monolayer from Molecular Dynamics Simulations. *Langmuir* **2007**, *23* (25), 12617–12623.

(2) Javanainen, M.; Lamberg, A.; Cwiklik, L.; Vattulainen, I.; Ollila, O. H. S. Atomistic Model for Nearly Quantitative Simulations of Langmuir Monolayers. *Langmuir* **2018**, *34* (7), 2565–2572.

(3) Menzl, G.; Dellago, C. Effect of Entropy on the Nucleation of Cavitation Bubbles in Water under Tension. *J. Chem. Phys.* **2016**, *145* (21), 211918.

(4) Wang, Z.-J.; Frenkel, D. Pore Nucleation in Mechanically Stretched Bilayer Membranes. *J. Chem. Phys.* **2005**, *123* (15), 154701.

(5) Sum, A. K.; Faller, R.; Pablo, J. J. de. Molecular Simulation Study of Phospholipid Bilayers and Insights of the Interactions with Disaccharides. *Biophys. J.* **2003**, *85* (5), 2830–2844.
